# Monosialotetrahexosylganglioside in the treatment of chronic oxaliplatin-induced peripheral neurotoxicity: TJMUCH-GI-001, a randomised controlled trial

**DOI:** 10.1016/j.eclinm.2021.101157

**Published:** 2021-10-29

**Authors:** Likun Zhou, Rui Liu, Dingzhi Huang, Hongli Li, Tao Ning, Le Zhang, Shaohua Ge, Ming Bai, Xia Wang, Yuchong Yang, XinYi Wang, Xingyun Chen, Zhiying Gao, Laizhi Luo, Yuanquan Yang, Xi Wu, Ting Deng, Yi Ba

**Affiliations:** a Tianjin Medical University Cancer Institute and Hospital, National Clinical Research Center for Cancer Tianjin's Clinical Research Center for Cancer, Key Laboratory of Cancer Prevention and Therapy Tianjin Medical University, Tianjin, China; b Medical Research Center, Peking Union Medical College Hospital, Chinese Academy of Medical Sciences and Peking Union Medical College, Beijing, China.; c Guangzhou Medical University, Guangzhou Chest Hospital, Guangzhou, China; d Division of medical oncology, the Ohio state university, Columbus, Ohio; e Cancer hospital, Chinese Academy of Medical Sciences and Peking Union Medical College, Beijing, China

**Keywords:** monosialotetrahexosylganglioside, GM1, oxaliplatin, neurotoxicity, CIPN, OIPN

## Abstract

**Background:**

Chronic oxaliplatin-induced peripheral neurotoxicity (OIPN) is the most troublesome and dose-limiting side effect of oxaliplatin. There is no effective treatment for chronic OIPN. We conducted a randomised controlled trial to investigate the efficacy of monosialotetrahexosylganglioside (GM1) in treating chronic OIPN.

**Methods:**

In this single-centre, double-blind, phase Ⅲ trial, gastrointestinal cancer patients with persistent chronic OIPN were randomised in 1:1 ratio to receive either GM1 or placebo at Tianjin Medical University Cancer Institute and Hospital, China. GM1 was dosed at 60 mg daily for every 3 weeks or 40 mg daily for every 2 weeks. Seven- and fourteen- day infusions were administered to concurrent oxaliplatin users and oxaliplatin discontinuation patients, respectively. The primary endpoint was the relief of neurotoxicity (≥30% improvement), measured by a newly developed patient reported outcome measure (MCIPN) based on prior questionnaires including the European Organization for Research and Treatment of Cancer Quality of Life Chemotherapy Induced Peripheral Neuropathy Questionnaire twenty-item scale. Visual analogue score (VAS) was used as another instrument for patients to evaluate the total Chronic OIPN treatment effect. VAS responders (≥30% improvement), double responders (≥30% improvement in both MCIPN and VAS), and high responders (≥50% improvement in the MCIPN total score) were also calculated. The secondary endpoints were safety and quality of life. The additional endpoints are progression-free survival (PFS), disease-free survival (DFS), overall survival (OS), and tumour response. (Trial registration number: NCT02486198 at ClinicalTrials.gov).

**Findings:**

Between May 2015 to December 2017, 145 patients were randomly assigned to receive either GM1 (n=73) and placebo (n=72). Majority of the patients in both arms (90% in GM1 and 83% in placebo) continued receiving oxaliplatin on the trial. More patients responded in the GM1 group than in the placebo group (MCIPN responders: 53% vs 14%, VAS responders: 49% vs 22%, double responders: 41% vs 7%, and high responders: 32% vs 13%, all *P* < ·01). Analyses were also performed in concurrent oxaliplatin users. The results were consistent with those of the whole group. No deleterious effects of GM1 on survival or tumour response were found. There were no ≥G3 GM1-related adverse events.

**Interpretation:**

In patients with chronic OIPN, the use of GM1 reduces the severity of chronic OIPN compared with placebo.

**Funding:**

This work was supported by clinical trial development fund of Tianjin Medical University Cancer Institute and Hospital (No.C1706).

**Research in context**

**Evidence before this study**

There was no effective treatment for chronic oxaliplatin-induced peripheral neurotoxicity (OIPN). Duloxetine was the only drug that showed benefit in treating post-chemotherapy neuropathic pain induced by oxaliplatin and paclitaxel. However, its effectiveness in non-painful chemotherapy-induced peripheral neurotoxicity or chemotherapy concurrent user has not been proven. Furthermore, 11% of patients discontinued duloxetine because of adverse events. Monosialotetrahexosylganglioside (GM1) is an active and safe agent to treat diabetic peripheral neuropathy and Parkinson's disease.

**Added value of this study**

The TJMUCH-GI-001 study showed the patients who received GM1 had a clinically meaningful improvement of chronic OIPN versus placebo. The chronic OIPN improvement was also seen in the oxaliplatin concurrent users which accounted for majority of the study patients. The response to GM1 treatment was fast onset and the improvement was detected as soon as after the first treatment cycle. More importantly, GM1 was well tolerated. There were no increased adverse events after adding GM1 to chemotherapy. To our knowledge, the TJMUCH-GI-001 study is the first randomised study demonstrating significant improvement of chronic OIPN with an agent in oxaliplatin concurrent users.

**Implications of all the available evidence**

There is a substantial unmet need for new therapies in chronic OIPN. The results of the TJMUCH-GI-001study show that GM1 can provide a notable improvement of chronic OIPN in oxaliplatin concurrent users, suggesting that GM1 could be a useful treatment option in patients with chronic OIPN.

Alt-text: Unlabelled box

## 1 Introduction

Oxaliplatin is a third-generation platinum complex which combined with other chemotherapy agents such as irinotecan, docetaxel, fluorouracil (5-FU), and 5-FU analogues, has become a central component of treatment regimen for gastrointestinal (GI) cancers in almost all disease phases in the neoadjuvant, adjuvant, perioperative, and palliative settings [1], [2], [3], [4], [5], [6], [7], [8].

Oxaliplatin-induced peripheral neurotoxicity (OIPN) is the most severe and dose-limiting toxicity of oxaliplatin-containing chemotherapy. OIPN is associated with high morbidity (> 85%) in both acute and chronic forms [7,[9], [10], [11]]. The National Cancer Institute Common Terminology Criteria for Adverse Events (NCI-CTCAE) grade 2 and 3 occurrences of chronic OIPN (OIPN) were 26–41% and 12–34%, respectively [6,7,11]. Nearly 50-70% of the patients experience ≥ grade 2 chronic OIPN [12,13]. Acute OIPN is often reversible during chemotherapy intervals. In contrast, chronic OIPN worsens over the treatment duration and, then certain number of chronic OIPN will last for years beyond the completion of chemotherapy. Indeed, in 84% and 69% of the patients, chronic OIPN lasted for about 2 years and 4 years, respectively, after the actual treatment [14,15]. The longest reported chronic OIPN duration was as long as 11 years [16].

Chronic OIPN usually affects patients who receive ≥ 540 mg/m^2^ of accumulative oxaliplatin [17]. It can occur in the early or later stages of chemotherapy, even after oxaliplatin cessation [18,19]. The occurrence of chronic OIPN was found to increase with increasing number of oxaliplatin-received cycles [10,13,20,21]. Compared with the sixth course, grade 3 chronic OIPN occurrence at the 12th oxaliplatin-treatment course tripled [13,20]. Furthermore, in clinical practice, more effective treatments (e.g., chemotherapy plus bevacizumab) cause more patients to develop chronic OIPN of ≥ grade 2 [12].

The typical extremital “stocking and glove” paresthesia and dysesthesia of chronic OIPN result in impairment of quality of life (QoL) and cause physical dysfunction, both during chemotherapy and after its cessation. Chronic OIPN also affects emotional functioning causing anxious/depressive symptoms and sleep disturbance [22,23]. Studies showed that 27–30% and 5–33% of the patients needed oxaliplatin dose reduction and withdrawal, respectively, because of chronic OIPN [11,[24], [25], [26], [27], [28]]. The fear of chronic OIPN even led colorectal cancer (CRC) patients to choose shorter adjuvant treatment durations at the cost of cure rate reduction [29].

The precise pathophysiologic mechanism of OPIN remains unclear. Acute OIPN is a Na^+^ channelopathy, which shows hyperexcitability of neurons and slows potential activation [30], [31], [32]. However, chronic OIPN is associated with not only reduced Na^+^ current but also oxaliplatin accumulation at the dorsal root ganglia (DRG) and spinal cord; therefore, causing DRG neurons atrophy and apoptosis [33], [34], [35].

Many attempts to reduce or prevent chronic OIPN have been made, including alternating chemotherapy regimens to reduce oxaliplatin cumulative dosage and using chemoprotectants. Unfortunately, no prophylactic agents have been proven effective in preventing chronic OIPN in phase III studies. Duloxetine was the only drug beneficial for the treatment of neuropathic pain caused by oxaliplatin and paclitaxel after chemotherapy; [36] however, its effect in non-painful OIPN has not been characterised. The “stop and go” is the most extensively studied non-pharmaceutical strategy, which achieved a longer OIPN-free interval in metastatic CRC but failed to reduce chronic OIPN [37]. Moreover, omitting oxaliplatin increased the risk of out-of-control cancer, when the disease still responded to oxaliplatin. Shortening the adjuvant chemotherapy duration for CRC from 6 to 3 months also failed to show non-inferiority in the IDEA study [13].

Since the most common events such as hematological toxicity and nausea/vomiting are now effectively managed, chronic OIPN has become the most survival-limiting adverse event (AE). The failure of chronic OIPN management has made it urgent to find a safe and effective agent for its treatment/prevention.

Monosialotetrahexosylganglioside (GM1) is an active agent for diabetic peripheral neuropathy (DPN) [38]. It has been approved by China Food and Drug Administration for vascular or traumatic central nervous system injury and Parkinson's disease. However, it was unavailable in North American and European Union. A ganglioside mixture (including GM1) improved DPN-related paresthesia in previous clinical trials [39], [40], [41]. Furthermore, GM1 elevates superoxide dismutase and glutathione levels to decrease anti-oxidant stress [42,43]. Animal studies showed that GM1 enhanced the activity of nerve growth factor, a protective factor of DRG [44]. To our knowledge, 3 studies (1 retrospective study and 2 randomised placebo-controlled trials) have evaluated the effectiveness of GM1 on chronic OIPN prevention [45], [46], [47]. However, the results were inconsistent.

Based on the aforementioned neuroprotective effects of GM1, we hypothesised that GM1 would ameliorate chronic OIPN. Therefore, we conducted a randomised phase Ⅲ trial, called the 001 trial of gastrointestinal oncology department of Tianjin Medical University Cancer Institute and Hospital (TJMUCH-GI-001), to investigate the efficacy of GM1 in treating chronic OIPN.

## 2 Methods

### 2.1 Patients

The GI cancer patients included in this study were of any age with persistent chronic OIPN, which was defined as experiencing daily paresthesia and/or dysesthesia during oxaliplatin-containing chemotherapy duration (including both chemotherapy duration and interval) for oxaliplatin concurrent user. For patients who stopped taking oxaliplatin, persistent chronic OIPN was defined as experiencing daily paresthesia and/or dysesthesia from the last dose of oxaliplatin (the duration should be more than 1 week and less than 4 weeks). Other eligibility criteria included adequate organ functions, and an Eastern Cooperative Oncology Group performance status (ECOG PS) of 0–2. Diabetics patients who had preexisted neuropathic symptoms (paresthesia and/or dysesthesia) before first oxaliplatin dose were excluded. Details of the other inclusion and exclusion criteria are presented in the Supplementary file. Informed consent was obtained from all the participants. This study was approved by the Institutional Review Board of Tianjin Medical University Cancer Institute and Hospital and registered at ClinicalTrials.gov (NCT02486198).

### 2.2 Study design

In this single-centre (Tianjin Medical University Cancer Institute and Hospital), double-blind (blind to both investigators and patients), phase Ⅲ trial, patients who have received at least one cycle of oxaliplatin infusion, were randomised in a 1:1 ratio to receive porcine brain-derived GM1 or identical-appearing placebo by intravenous infusion. GM1 and placebo were obtained from Qilu Pharmaceutical Co., Ltd, Shandong, China. The urn model of randomisation was applied, and the allocation procedure was masked. The investigators generated the random allocation sequence by blindly choosing one coloured ball from two different coloured balls once in a dark box (the numbers of the two coloured balls vary according to the method of urn model). One investigator (Zhou) assigned participants to interventions according to the allocation sequence. Considering that the ultracentrifugable platinum elimination of oxaliplatin was of 7·15 days and the clinical practicality [48], seven consecutive infusions of the study drug in each chemotherapy cycle corresponded to the longest acceptable regimen administration for concurrent oxaliplatin users after face-to-face communication with the patients. The treatment plan is shown in supplementary Fig. S1. Briefly, the patients who remained on oxaliplatin at the time of enrollment received concurrent once daily placebo or GM1 intravenous infusions for 7 consecutive days (day 1 to day 7) with each chemotherapy cycle. The first dose of study drug must be infused before oxaliplatin. For patients who was not on oxaliplatin at time of enrollment, placebo or GM1 was given intravenously once daily for 14 consecutive days in every 14-day cycle. For patients who ceased oxaliplatin after enrollment, the treatment scheduled was changed to consecutive daily infusions for 14 days in every 14-day cycle as maintenance. Each 7 consecutive days study drug infusion with each oxaliplatin-chemotherapy cycle or each 14 consecutive days study drug infusion in oxaliplatin discontinuer was recorded as one treatment cycle. Referring to dose and schedule of GM1 used for Parkinson's disease and central nervous system injury in drug instruction, GM1 was dosed at 60 mg daily for every 3 weeks or 40 mg daily for every 2 weeks (weekly intensity was 20 mg daily) and the maximum GM1 therapy duration was 126 times (18 weeks) according to the GM1 instruction. (GM1 drug administration instruction is listed in Supplementary file)

Treatment was discontinued if any of the following conditions were met: (1) chronic OIPN progression defined as modified chemotherapy induced peripheral neuropathy questionnaire (MCIPN) total scores or visual analogue scale (VAS) showed ≥ 30% deterioration compared with baseline; (2) patients refused further treatment; (3) non-myelosuppressive serious AEs attributed to GM1. All subjects were followed up after drop-out from the trial. The follow-up frequency was once every 12 weeks by telephone.

### 2.3 Outcome measures

The European Organization for Research and Treatment of Cancer Quality of Life Chemotherapy Induced Peripheral Neuropathy Questionnaire twenty-item scale (EORTC QLQ‐CIPN20) was one of the most widely accepted tools for chemotherapy-induced peripheral neuropathy (CIPN) assessment. Because some items were not suitable for patients receiving oxaliplatin and no Chinese version of EORTC QLQ-CIPN20 at the onset of our clinical trial, a patient reported outcome measure based on prior questionnaires including the EORTC CIPN20 were made as MCIPN, with most items the same as EORTC QLQ-CIPN20, to meet the characteristics of oxaliplatin after face-to-face interviews with patients. It addressed sensory symptoms in the upper (six questions) and lower extremities (seven questions), temperature sensation (one question), and hearing sensation (one question). It also addressed motor and autonomic functions with five and three questions, respectively. A linear scale was applied to each question [23,49]. Questions were scored from 0–10 (0 = not at all; 10 = as bad as it can be) and the scores were summed (rang 0-230). VAS was used as another instrument for patients to evaluate the total chronic OIPN treatment effect; it was a linear analogue scale from -10 to 10 (-10 = double progress; 0 = no change; 10 = complete relieve). The details of EORTC QLQ‐CIPN20 modification reasons, MCIPN items, and VAS are listed in the Supplementary file.

At baseline (within the 72 hours before study drugs infusion after enrolled), chronic OIPN was assessed using two questionnaire tools (MCIPN, and NCI-CTCAE v4·03). Then, for oxaliplatin concurrent user, all three questionnaire tools (all MCIPN items, VAS and NCI-CTCAE v4·03) were measured within 72 hours before each GM1 infusion cycle. For patients who stopped taking oxaliplatin, all the three questionnaire tools were measured every 14 days after patients enrolled. All patients who dropped out without chronic OIPN progression were also assessed chronic OIPN (all the three questionnaire tools) after the last treatment cycle. Acute OIPN was assessed using 11-item questionnaires (yes/no response format) for 5 days after oxaliplatin administration of each cycle of chemotherapy [9,50]. The medical outcomes study item short from health survey (SF-36) questionnaires were assessed to evaluate QoL before first treatment cycle, then every 6 weeks and after the last treatment cycle.

AEs were evaluated according to NCI-CTCAE v4·03 during and after the treatment completion.

### 2.4 Endpoints

The primary endpoint was the relief of neurotoxicity (i.e. MCIPN responder). It was defined as ≥30% improvement in MCIPN total score compared to the baseline at any point and such improvement was durable until end of the study. Visual analogue score (VAS) was used as another instrument for patients to evaluate the total chronic OIPN treatment effect. Thirty percent improvement was considered clinically significant based on prior CIPN and pain-related studies [51], [52], [53]. Item responders were defined as patients who had ≥ 30% improvement in corresponding MCIPN item scores. Double responders were defined as patients who had ≥ 30% improvement in both MCIPN and VAS scores. High responders were defined as patients who had ≥ 50% improvement in MCIPN total scores (compared with the baseline scores). ^36,51^The secondary endpoints were safety and quality of life. Because whether GM1 has influence on tumour is unclear, additional endpoints, including progression-free survival (PFS), disease-free survival (DFS), overall survival (OS) and tumour response (measured by Response Evaluation Criteria in Solid Tumors, RECIST Version 1·1), were also measured as additional endpoints. Tumour response was assessed by each investigator.

### 2.5 Statistical analysis

As the natural history of chronic OIPN in Chinese has not been well described, we first followed 79 patients in our department who experienced persistent chronic OIPN from 2014 to 2015 by telephone. Only 2·5% of these patients had any kind of sensory neurotoxicity relief within the first 3 months after oxaliplatin. We assumed at least 30% responders, which was considered clinically meaningful, in the GM1 group and 3·0% in the placebo group. The sample size calculation showed that 144 patients (72 each arm) were required, with alpha and beta errors of 0·05 and 0·2, respectively.

Progression-free survival (PFS), disease-free survival (DFS), and overall survival (OS) were compared between the treatment arms using a log-rank test with Kaplan-Meier curves. Chi-square tests were used to test the differences in proportions, and Student's t-test was used to compare continuous variables. Risk ratio and hazard ratios with their 95% confidence intervals were calculated. A two-tailed *P* < ·05 was considered statistically significant.

The Consolidated Standards of Reporting Trails (CONSORT) checklist was listed in the supplementary file and this randomised trial was adherent to CONSORT.

### 2.6 Role of the funding source

This study was funded by clinical trial development fund of Tianjin Medical University Cancer Institute and Hospital (No.C1706). The funding had no role in the study design or the data collection, analysis, and data interpretation, or in the writing of the manuscript, and in the decision to publish the results.

## 3 Results

### 3.1 Patients

**Fig. 1:**
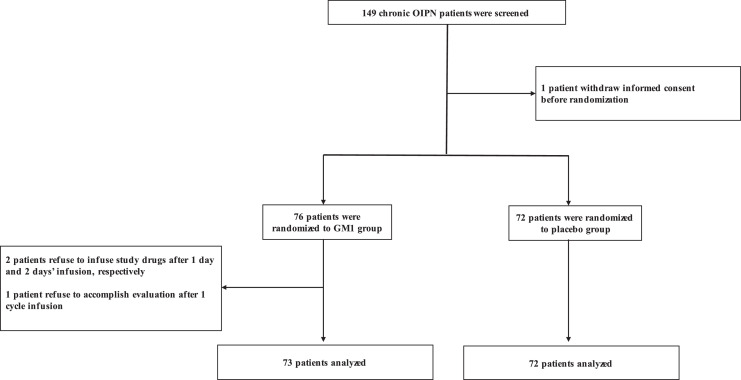
Study flow-chart. OIPN, oxaliplatin-induced peripheral neurotoxicity; GM1, monosialotetrahexosylganglioside.

From May 2015 to December 2017, 149 consecutive eligible cases were screened. One patient withdrew consent before randomisation. One patient refused to fill out questionnaires after one cycle infusion, although she declared chronic OIPN improvement without discomfort. Two other patients refused to infuse the study drugs after 1 day and 2 days of infusion without AEs, respectively. Finally, 73 patients in the GM1 group and 72 in the placebo group, who received at least one cycle of the study drug, were analysed for efficacy and safety (Fig. 1). Among them, sixty-six and sixty patients received concurrent oxaliplatin in GM1 group and placebo group, respectively.

All baseline characteristics were balanced except that the MCIPN score was higher in the GM1 group. The details of patient baseline characteristics are summarised in Table 1.

**Table 1:** Characteristics of the study cohorts

Characteristics	GM1 group (N=73)	Placebo group (N=72)
Age (median, range)	60 (23-79)	60 (24-75)
Male- (n, %)	52 (71.2)	45 (62.5)
Diagnosis (n, %)		
Esophageal cancer	1 (1.4)	1 (1.4)
Stomach cancer	24 (32.9)	19 (26.4)
Colon and rectal cancer	43 (58.9)	47 (65.3)
Pancreatic cancer	2 (2.7)	3 (4.2)
Biliary and ampulla cancer	2 (2.7)	1 (1.4)
Primary site unknown	1 (1.4)	1 (1.4)
Stage (n, %)		
Ⅰ	0 (0)	2 (2.8)
Ⅱ	8 (11)	6 (8.3)
Ⅲ	14 (19.2)	21 (29.2)
Ⅳ	49 (67.1)	38 (52.8)
Local advanced	0 (0)	1 (1.4)
Unknown	2 (2.7)	4 (5.6)
Cumulative oxaliplatin dose (mg/m^2^, median, range)	680 (65-1105)	665 (85-1275)
Baseline neurotoxicity scores*	21 (4-112)	16 (3-70)
Time from persistent COIPN diagnosis to receive the study drug (mean, range, month)	0.96 (0-3.0)	0.72 (0-4.8)
Concurrent oxaliplatin user (n, %)	66 (90)	60 (83)

COIPN, chronic oxaliplatin-induced peripheral neurotoxicity; GM1, monosialotetrahexosylganglioside;

⁎ *P* < .05

### 3.2 Oxaliplatin-induced peripheral neurotoxicity

Because MCIPN had not been validated, we first tested the correlation of baseline MCIPN scores with NCI-CTCAE neuropathy grades and SF-36 scores. Marked differences were detected in MCIPN scores between NCI-CTCAE grade 3 and grade 2 (median: 31 vs. 18 scores, *P* <·05). Furthermore, the negative association between baseline MCIPN scores and SF-36 scores was strong (r = -0·514, *P* < ·001) (Supplementary Fig. S2), whereby patients with higher MCIPN scores had lower SF-36 scores, which meant lower QoL.

The mean GM1/placebo treatment cycles were 2·2 (range, 1–6) in the GM1 group and 1·8 (range 1–5) in the placebo group (*P* > ·1). There were more patients measured as MCIPN, VAS, double, and high responders in the GM1 group than in the placebo group (MCIPN responders: 53% vs. 14%, *P* < ·0001; VAS responders: 49% vs 22%, *P* = ·001; double responders: 41% vs. 7%, *P* < ·0001; high responders: 32% vs. 13%, *P* = ·004). Using NCI-CTCAE as the assessment standard, more patients in the GM1 group showed a neurotoxicity improvement of ≥ 1 grade but without statistical significance. However, there was no significant difference between the two groups in patients who had at least one symptom of acute OIPN (56% in GM1 group vs 49% in placebo group, *P* = ·44). The results are summarised in Table 2. During therapy, 19/73 in the GM1 group and 35/72 in the placebo group experienced chronic OIPN progression (*P* = ·005). After having achieved planned oxaliplatin cycles, 20 and 9 patients transitioned over to maintenance in GM1 group and placebo group, respectively. Among the 20 patients who transitioned over to maintenance in GM1 group, 15 were responders and the other 5 were non-responders. In contrast, two and seven patients were responders and non-responders in placebo group, respectively.

**Table 2:** Effectiveness of GM1 for COIPN

Arm /Measure	MCIPN Responder n (%)	VAS Responder n (%)	Double Responder^a^ n (%)	High Responder^b^ n (%)	NCI-CTCAE (improve ≥ 1grade)		AOINP in oxaliplatin concurrent user
					Peripheral sensory n (%)	Peripheral motor n (%)	n (%)
GM1 (N=73)	39 (53)	36 (49)	30 (41)	25 (32)	8 (11)	3 (5)	37 (56)
Placebo (N=72)	10 (14)	16 (22)	5 (7)	9 (13)	5 (7)	3 (4)	29 (49)
RR	3.85	2.22	5.92	2.74	0.97	1	1.14
95% CI	2.08-7.11	1.36-3.63	2.43-14.40	1.38-5.46	0.88-1.07	0.94-1.07	0.81-1.60
P value	<0.0001	0.001	<0.0001	0.004	0.56	0.99	0.44

COIPN, chronic oxaliplatin-induced peripheral neurotoxicity; AOIPN, acute oxaliplatin-induced peripheral neurotoxicity; GM1, monosialotetrahexosylganglioside; RR, risk ratio; CI, confidence interval; NCI-CTCAE, The National Cancer Institute Common Terminology Criteria for Adverse Events; MCIPN, modified chemotherapy induced peripheal neuropathy questionaire; VAS, visual analogue scale.

a Double responders were defined as patients who experienced 30% improvement of both MCIPN and VAS.

b Responders and high responders were defined as patients who experienced 30% and 50% improvement, respectively.

To further elucidate the effectiveness of GM1 in chronic OIPN, analysis was performed for concurrent oxaliplatin users and post oxaliplatin users. The results were consistent with those of the whole group (Supplementary Table S1 and Table S2). In additional, more patients in GM1 group benefited after each cycle of study drug (Supplementary Fig. S3). Consistently, more patients benefited from GM1 with accumulative treatment cycles (Supplementary Fig. S4). During therapy, 18/66 in the GM1 group and 30/60 in the placebo group experienced chronic OIPN progression among concurrent oxaliplatin users (*P* = ·009).

**Fig. 2:**
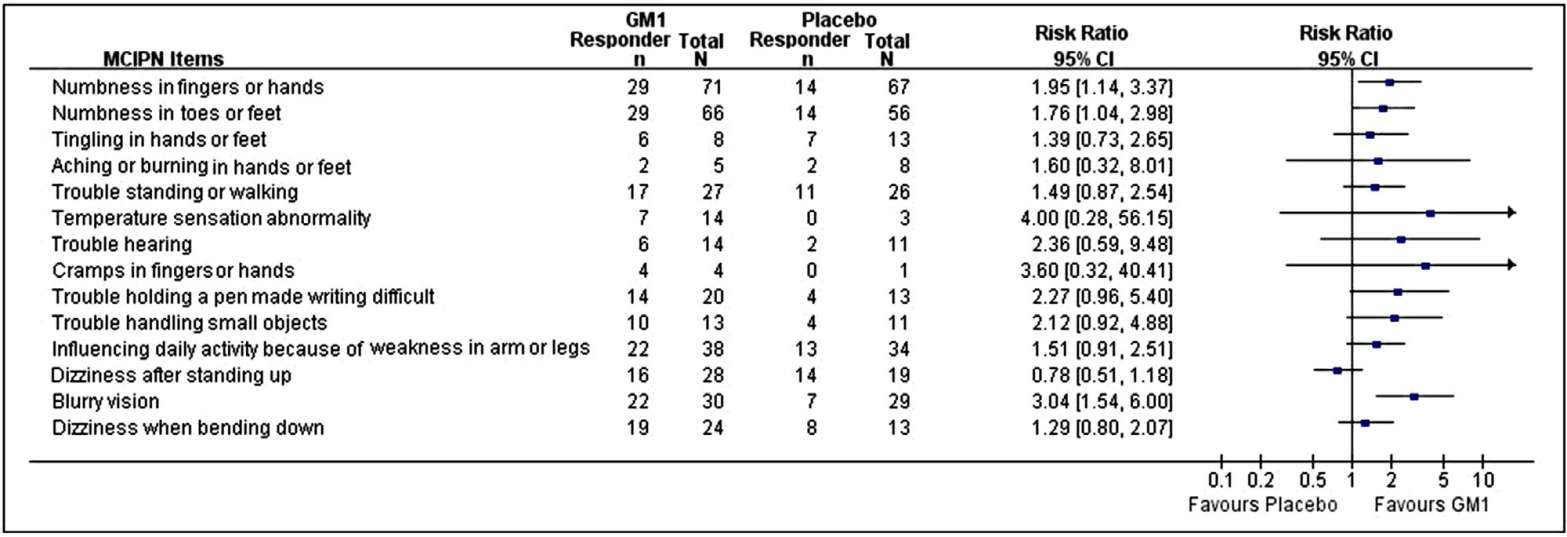
MCIPN item analysis MCIPN, modified chemotherapy induced peripheal neuropathy questionaire; GM1, monosialotetrahexosylganglioside; CI, confidence interval. Responders were defined as patients who had ≥ 30% improvement in corresponding MCIPN item scores.

The MCIPN items analysis showed that the improvements in numbness and blurry vision were in favour of GM1. The trends in the improvements of the other items, except for dizziness after standing up, were in favour of GM1 (Fig. 2). The improved scores of each MCIPN items were also listed in the supplementary file (Fig. S5).

Some patients reported hypogeusia, which may be a symptom of neuropathy and results in loss of appetite [54]. At baseline, 58 out of 88 assessed patients reported hypogeusia with a median 32·5% taste loss (range, 5–80%). Although more patients who received oxaliplatin had complete hypogeusia relief in the GM1 group, there was no significant difference (9/29 vs 7/29).

### 3.3 Survival and safety

**Fig. 3:**
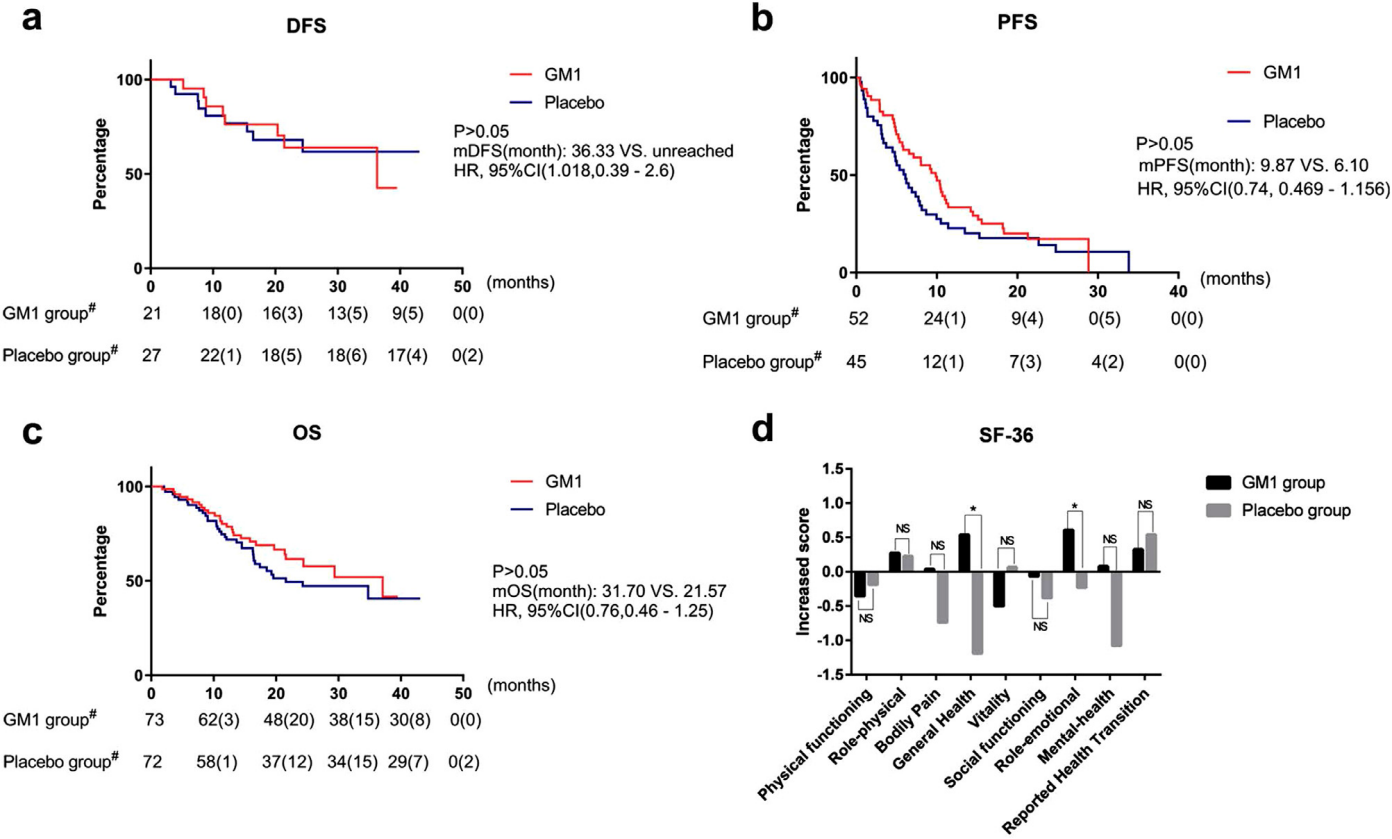
Survival and quality of life (a) DFS; (b) PFS; (c) OS; (d) SF-36 DFS, disease-free survival refers to the percentage of participants who have not experienced a recurrence of tumour or mortality at any given time; PFS, progression-free survival refers to the percentage of participants who have not experienced a progression of tumour or mortality at any given time; OS, overall survival refers to the percentage of participants who have not experienced a mortality at any given time; GM1, monosialotetrahexosylganglioside; HR, hazard ratio; CI, confidence interval; SF-36, the medical outcomes study item short from health survey; NS, not statistically significant. # denotes censured individual numbers were listed in brackets and * P < •05

The median follow-up period was 16·6 months (0·8–43·1 months) as of December 2018. Four patients were lost to follow-up. There were no deleterious effects of GM1 on survival (DFS, PFS, and OS, Fig. 3a-c). During therapy, seven and eight patients in the GM1 and placebo groups, respectively, experienced tumour progression.

AEs were listed in Table 3. The most frequent grade 3 or 4 AEs, including neutropenia and hypoleukemia, were not significantly different between the two groups. None of the AEs were related to GM1. Daily infusion inconvenience was the main reason for the discontinuation of the study drugs infusion after reaching the planned oxaliplatin chemotherapy cycle (52/73 in the GM1 group and 34/72 in the placebo group).

**Table 3:** Adverse events

	GM1 (n=73)		Placebo (n=72)	
Adverse events	1-2 grade	3-4 grade	1-2 grade	3-4 grade
Neutropenia	29	8	26	4
Leucopenia	22	4	18	1
Thrombocytopenia	31	0	23	0
Anemia*	39	0	26	3
Anorexia	3	2	2	0
Nausea	18	2	9	0
Vomiting	14	1	9	0
Appendicitis	0	1	0	0
Infusion related reaction	4	0	3	0
Hand-Foot Syndrome	0	0	1	2
Intestinal obstruction	0	0	1	2
Urinary tract infection	0	0	0	1
Hypercalcemia	1	2	2	2
Hypertension	0	1	0	1
AKP increased	48	0	37	1
Fever	2	0	0	0
Lung infection	0	0	1	0
Diarrhea	1	0	2	0
Ischemia cerebrovascular	2	0	0	0
Proteinuria	11	1	10	0
Hyperbilirubinemia	9	1	7	0
Hypokalemia	2	1	1	0
Hypoalbuminemia	35	0	29	0
AST increased	30	0	31	0
ALT increased*	9	0	19	0
LDH increased	20	0	22	0
Hyperglycemia	16	0	11	0
Creatinine increased	8	0	4	0
Hemoglobinuria	6	0	7	0
Hypoalbuminemia	4	0	4	0
Hypoglycemia	2	0	5	0
Hypernatremia	1	0	0	0
Hypocalcemia	0	0	2	0
Dyspnea	1	0	1	0
Malaise	1	0	0	0
Headache	1	0	0	0
Palpitations	1	0	1	0
Abdominal distension	0	0	1	0

GM1, monosialotetrahexosylganglioside

⁎ *P* < •05

### 3.4 Quality of life

QoL analysis was carried out on the data from 86 patients. The SF-36 questionnaire submission rates were acceptable (GM1 group: 63% vs placebo group: 55%, *P* = ·36). The results showed that two out of eight domains of health (general health and role-emotional) in the GM1 group were improved when compared with those in placebo group (Fig. 3d).

## 4 Discussion

Chronic OIPN is the most troublesome and dose-limiting side effect of oxaliplatin. It reduces the number of chemotherapy cycles and shortens the disease control duration. The years-long lasting chronic OIPN, following treatment in certain number of patients, worsens patient’ QoL and causes psychological problems. In the age of no effective drugs for chronic OIPN, medical decision has to balance the benefit of oxaliplatin continuation and the risk of neuropathy. “Stop and go” strategy and reducing chemotherapy cycles were the most widely investigated nonpharmacological approaches to reduce chronic OIPN. However, both the approaches are of limited success. While both these approaches have been studied in CRC, whether they can be likewise applied to other cancers remains unclear. Nevertheless, 6 months oxaliplatin-containing chemotherapy is still the standard as adjuvant therapy for gastric cancer, pancreatic cancer, and most CRCs [3,55]. Oxaliplatin-based chemotherapy should be prescribed as much as possible in advanced digestive tract tumours when the cancer still responds to treatment. For advanced CRC patients, the median cycle of FOLFOX as first-line chemotherapy was 11–12 cycles [6,56]. Thus, chronic OIPN was inevitable in most advanced and curative GI cancer cases.

Before our study proved that GM1 was effective in chronic OIPN treatment, many agents have been tried in chronic OIPN. Glutathione, goshajinkigan, and neurotropin showed effectiveness in preventing OIPN in small-sample randomised controlled trials [57], [58], [59]. However, chronic OIPN still progressed with increasing number of chemotherapy cycles when using glutathione [57]. The role of MR309, a sigma-1 receptor antagonist, needs further investigation [60]. Duloxetine, an antidepressant drug, was the only drug for painful CIPN treatment. Although it seemed that duloxetine was also effective in treating numbness and tingling in the feet, this drug was studied in painful CIPN patients who had completed chemotherapy. The effectiveness and safety of duloxetine have not been tested in patients receiving oxaliplatin. Furthermore, antidepressants are associated with many AEs. In this phase Ⅲ study, 11% of patients discontinued duloxetine because of AEs [36]. Another study, which used duloxetine to treat OIPN, showed that 23·1% of the patients prematurely withdrew within the first 3 weeks of 12 weeks’ treatment regimen [61]. A cross-sectional study of 406 patients found unexpected failure of OIPN management in adjuvant CRC patients from 2016 to 2019 at 16 French centers in the post-duloxetine era [62]. The inhibitory effect of duloxetine on cytochrome P450 is another issue that needs to be considered when it is combined with other agents.

The “coasting phenomenon”, defined as patients who experience more severe OPIN in the first 3–4 months after oxaliplatin cessation, is a particular characteristic of oxaliplatin. Based on the updated data, we hypothesised to explain the coasting phenomenon. Red blood cell (RBC)-bound oxaliplatin accounts for nearly 40% of the total oxaliplatin and erythrocytic platinum's elimination half-life is close to that of RBC's [63]. With the natural history of RBC clearance, which has a life-span of about 120 days, oxaliplatin may constantly release from the broken RBCs. The oxaliplatin, which released from broken RBC, may cause OIPN directly or hamper DRG oxaliplatin clearance. This was indicated by the fact that perioperative haemolysis following liver surgery could induce plasma platinum elevation and worsen OIPN [64]. If this hypothesis holds true, prolonged drug prescription is the key in chronic OIPN treatment and prophylaxis. This may be the reason why GM1 and other agents failed to prevent chronic OIPN in phase Ⅲ studies [47]. In the phase Ⅲ study of prevention chronic OIPN by GM1, 80 mg GM1 was administrated from day 0 to day 4. This study found that GM1 could reduce acute OIPN while it failed to reduce chronic OIPN. Considering the total dose of this study was higher than that in our study and the ultracentrifugable platinum elimination of oxaliplatin was of 7·15 days, the shorter GM1 use duration may be the cause of failure in prevention chronic OIPN of this study [47]. However, 2 other study (1 retrospective study and 1 randomised placebo controlled trial) showed GM1 can effectively prevent both chronic OIPN and acute OIPN [45,46]. These inconsistent results of GM1 in OIPN prevention suggested that more well-designed trails and/or patients based Meta-analysis are needed.

Because no drug has been effective in preventing chronic OIPN, it is important to address when GM1 should be administered. The cumulative oxaliplatin doses at enrollment were 680mg/m^2^ and 665mg/m^2^ in GM1 group and placebo group, respectively. Both doses are nearly equal to 8 cycles biweekly oxaliplatin treatment (85mg/m^2^ oxaliplatin for each biweekly treatment regimen). Moreover, our study showed that persistent chronic OIPN was a feasible indicator for treatment. It is an easily understood criterion that can avoid discrepancies between clinicians and patients. In our study, 91·7% of the patients with persistent OIPN were NCI-CTCAE grade 2, a proper grade to begin medical intervention. Because nearly half of the patients would not develop ≥ grade 2 chronic OIPN, treating patients with persistent chronic OIPN is more economical than prevention [12,13].

Good safety is another important advantage of GM1. It had no impact on tumour response and patient survival. Moreover, the long-term (16 weeks, 18 weeks, and 5 years) safety of GM1 has been proven in patients with Parkinson's disease [65], [66], [67]. There were no GM1 related grade 3–4 AEs in our study or other studies [46,47,68]. Because the metabolism of GM1 is independent of cytochrome P450, its long-term use alone or in combination with other agents such as duloxetine seems to be a reasonable option.

To adapt to the characteristics of oxaliplatin, EORTC QLQ‐CIPN20 was modified as MCIPN with most items identical to that in EORTC QLQ‐CIPN20. The results also showed that MCIPN scores correlated with NCI-CTCAE grades and patients’ QoL. Although MCIPN had not been validated, these results showed that it can be used in chronic OIPN assessment.

Our items analysis showed that GM1 benefited 3 of all 14 items (numbness in fingers or hands, numbness in toes or feet and blurry vision). Most other items showed the trends favour of GM1 group. The reason may be that we enrolled patients who were experiencing daily paresthesia and/or dysesthesia and only few patients had symptoms other than numbness. The test powers were not enough to evaluate these differences.

Despite our impressive results, we are aware that our study raises some questions. (1) The optimal dosage and treatment duration of GM1 as a therapy for chronic OIPN are still unknown. Whether a higher dosage and/or longer prescription will bring additional benefits are still unknown; (2) Multivariate logistic regression showed that the time from persistent chronic OIPN diagnosis to receiving the study drug was an influencing factor of efficacy (*P* = ·01, data not shown). Based on the fact that the time from chronic OIPN diagnosis to receive study drug were 0-3 months in GM1 group and 0-4.8 months in the placebo group (Table 1), whether longer- existing chronic OIPN will benefit from GM1 is also unclear; (3) The inconvenience of 7-day infusion was the main reason behind patient drop-out after their planned chemotherapy cycles were achieved. It reduced study drug treatment cycles. Although daily dosing scheme of GM1 for 1 week reduced its feasibility, higher dose and longer duration (100 mg, day 0 - day 14) was applied by another ongoing clinical trials (NCT04395339). Future studies shall focus on the optimal GM1 dosage and medication regimen optimization.

In summary, our results demonstrated that exogenously administered GM1 reduces the severity of chronic OIPN compared with placebo.

## Contributors

Likun Zhou: study design, data collation, patient's follow-up, data analysis, data interpretation, patient's enrollment, article writing, assigned participants to interventions, accessed and were responsible for the raw data associated with the study

Rui Liu: study design comments, data collation, patient's enrollment

Dingzhi Huang: study design, data interpretation, article writing comment

Hongli Li: study design comments, patient's enrollment, article writing comment

Tao Ning: patient's enrollment, article writing comment

Le Zhang: patient's enrollment, article writing comment

Shaohua Ge: patient's enrollment, article writing comment

Ming Bai: patient's enrollment, article writing comment

Xia Wang: patient's enrollment, article writing comment

Yuchong Yang: patient's enrollment assistant, article writing comment

XinYi Wang: patient's enrollment assistant

Xingyun Chen: patient's enrollment assistant

Zhiying Gao: data analysis, data interpretation, article writing

Laizhi Luo: data collation

Yuanquan Yang: article writing, data analysis

Xi Wu: article writing comment

Ting Deng: article writing comment

Yi Ba: study design, data interpretation, article writing, accessed and were responsible for the raw data associated with the study

## Data sharing statement

All the participant data and additional data including (study protocol, statistical analysis plan, informed consent form) are available by sending requirement to e-mail: zhoubaling123@163.com from 2021-9-30 to 2026-9-30.

## Funding

This work was supported by clinical trial development fund of Tianjin Medical University Cancer Institute and Hospital (No.C1706).

## Declaration of Competing Interest

All authors declare no competing interests.

## Appendix Supplementary materials

Image, application 1

Image, application 2
