# Supplementary material for: Monosialotetrahexosylganglioside in the treatment of chronic oxaliplatin-induced peripheral neurotoxicity: TJMUCH-GI-001, a randomised controlled trial
Source: eClinicalMedicine. 2021 Oct 29;41:101157. doi: 10.1016/j.eclinm.2021.101157 (PMC8569480; doi:10.1016/j.eclinm.2021.101157)
Supplement: Supplementary file 2 [file mmc2.docx]

SUPPLEMENTARY APPENDIX

Table of Contents

[Investigators 2](#_Toc80573119)

[Inclusion and exclusion criteria 3](#_Toc80573120)

[The reasons of European Organization for Research and Treatment of Cancer Quality of Life‐Chemotherapy‐Induced Peripheral Neuropathy Questionnaire (EORTC QLQ‐CIPN20) modification 4](#_Toc80573121)

[Modified chemotherapy induced peripheral neuropathy questionnaire (MCIPN) items 6](#_Toc80573122)

[Visual Analogue Scale (VAS) 7](#_Toc80573123)

[Fig S1. Administration design 8](#_Toc80573124)

[Fig S2. Close correlation between MCIPN and SF-36 at baseline (145 samples) 9](#_Toc80573125)

[Fig S3. Responders in oxaliplatin concurrent user after each treatment cycle 10](#_Toc80573126)

[Fig S4. Responders in oxaliplatin concurrent user with accumulative treatment cycles 11](#_Toc80573127)

[Fig S5. The improved scores of each MCIPN items 12](#_Toc80573128)

[Table S1. Efficacy of GM1 for chronic OIPN in concurrent oxaliplatin users 13](#_Toc80573129)

[Table S2. Efficacy of GM1 for chronic OIPN in post oxaliplatin users 14](#_Toc80573130)

[GM1 instruction 15](#_Toc80573131)

# Investigators

Likun Zhou, MD ^1^, Rui Liu, MD ^1^, Dingzhi Huang, MD ^1^, Hongli Li, MD ^1^,Tao Ning, MD ^1^,Le Zhang, MD ^1^, Shaohua Ge, MD ^1^, Ming Bai, MD ^1^, Xia Wang, MD ^1^, Yuchong Yang, MD ^1^, XinYi Wang, MD ^1^, Xingyun Chen, MD ^1^,Zhiying Gao, MD ^2^, Laizhi Luo, MD ^3^,Yuanquan Yang, MD ^4^,Xi Wu, MD ^5^,Ting Deng, MD ^1^, Yi Ba MD, Ph.D ^,1^*

1 Tianjin Medical University Cancer Institute and Hospital, National Clinical Research Center for Cancer Tianjin's Clinical Research Center for Cancer, Key Laboratory of Cancer Prevention and Therapy Tianjin Medical University, Tianjin,China.

2 Medical Research Center, Peking Union Medical College Hospital, Chinese Academy of Medical Sciences and Peking Union Medical College, Beijing, China.

3 Guangzhou Medical University, Guangzhou Chest Hospital, Guangzhou, China.

4 Division of medical oncology, the Ohio state university, Columbus, Ohio

5 Cancer hospital, Chinese Academy of Medical Sciences and Peking Union Medical College, Beijing, China

* Corresponding author e-mail：bayi@tjmuch.com

# Inclusion and exclusion criteria

Inclusion criteria: (1) Patients of any age with persistent chronic oxaliplatin-induced peripheral neurotoxicity (OIPN), defined as experiencing paresthesia and/or dysesthesia every day during oxaliplatin-containing chemotherapy or ceased oxaliplatin within the last 4 weeks; (2) Histologically verified gastrointestinal (GI) cancer; (3) Hematological, hepatic, and renal functions should be adequate; (4) Eastern Cooperative Oncology Group (ECOG) performance status of 0–2.

Exclusion criteria included: (1) Previous or concurrent drugs for oxaliplatin-induced peripheral neurotoxicity (OIPN) prevention or treatment; (2) Patients with other neurologic diseases or neuropathies caused by other diseases such as diabetes mellitus; (3) Patients with brain metastasis-related symptoms; (4) Chemotherapy regimen included other neurotoxic agents, such as taxoid, other platinum or vinca alkaloid; (5) Patients with family history of a familial neuropathy; (6) Patients unable to comply with the protocol.

# The reasons of European Organization for Research and Treatment of Cancer Quality of Life‐Chemotherapy‐Induced Peripheral Neuropathy Questionnaire (EORTC QLQ‐CIPN20) modification

1. Although the symptoms are generally symmetric, they are not identical between the left and right sides. A certain number of patients stated that they could not precisely score their symptoms if left and right hands/feet were evaluated together. Another study, which assessed the sensory and motor response of OIPN, also showed differences between the left and right hands.^1^ Thus, tingling, numbness, and aching or burning pain were separately evaluated by left and right hand/foot.

2. All the patients in our department were informed to avoid touching cold things that would exacerbate or trigger the neuropathy at a low cumulative oxaliplatin dose. The item of *trouble distinguishing temperature of hot and cold water* was changed as *temperature sensation abnormality*. Patients were asked whether they experienced differences when touching things such as hot water. A score of 0 was allotted if the sensation remained unchanged, while 10 was allotted if the patients could not feel temperature changes (for example, cannot feel hot when they use hot water to wash feet).

3. Cramps were rarely observed in OIPN. We did not find hand cramps and only two patients reported foot cramps in the previous follow-up of the 79 patients. The hand cramps were removed, and cramps of the feet were evaluated separately by left feet/toe and right feet/toe.

4. In order to be more practical, the following three items were combined as *influencing daily activities because of weakness in the arms and/or legs:* Trouble opening a jar/bottle due to loss of strength in hands? Trouble walking because your feet come down to hard? and Trouble walking stairs or standing up from a chair due to weakness in legs?

5. Like other studies^2,3^, the following two items were excluded because only a few patients would answer the questions: Only for those driving cars: Trouble driving due to use of pedals? and only for males: Trouble getting or maintaining an erection?

6. When we communicated with the patients and questioned the item of dizziness after standing up, some patients addressed they experience dizziness when bending down, which is also a symptom of autonomic abnormality. Therefore, the item of *dizziness when bending down* was added.

1. Tabata A, Kanai M, Horimatsu T, et al: Changes in upper extremity function, ADL, and HRQoL in colorectal cancer patients after the first chemotherapy cycle with oxaliplatin: a prospective single-center observational study. Support Care Cancer 26:2397-2405, 2018

2. Smith EML, Knoerl R, Yang JJ, et al: In Search of a Gold Standard Patient-Reported Outcome Measure for Use in Chemotherapy- Induced Peripheral Neuropathy Clinical Trials. Cancer Control 25:1073274818756608, 2018

3. Le-Rademacher J, Kanwar R, Seisler D, et al: Patient-reported (EORTC QLQ-CIPN20) versus physician-reported (CTCAE) quantification of oxaliplatin- and paclitaxel/carboplatin-induced peripheral neuropathy in NCCTG/Alliance clinical trials. Support Care Cancer 25:3537-3544, 2017

| Modified chemotherapy induced peripheral neuropathy questionnaire (MCIPN) items |
| --- |
| Sensory scale |
| Left hand tingling in left fingers or hands? |
| Right hand tingling in right fingers or hands? |
| Left foot/toe tingling in left toes or feet? |
| Right foot/toe tingling in right toes or feet? |
| Left hand numbness in left fingers or hands? |
| Right hand numbness in right fingers or hands? |
| Left foot/toe numbness in left toes or feet? |
| Right foot/toe numbness in right toes or feet? |
| Left hand/fingers aching or burning pain |
| right hand/fingers aching or burning pain |
| Left foot/toes aching or burning pain |
| Right foot/toes aching or burning pain |
| Trouble standing or walking? |
| Temperature sensation abnormality |
| Trouble hearing |
| Motor scale |
| Left fingers/hand cramps in left fingers/hand |
| Right fingers/hand cramps in right fingers/hand |
| Trouble holding a pen which made writing difficult? |
| Trouble handling small objects (eg, buttoning a blouse)? |
| Influencing daily activity because of weakness in arm/legs |
| Autonomic scale |
| Blurry vision |
| Dizziness after standing up |
| Dizziness when bending down |

# Visual Analogue Scale (VAS)


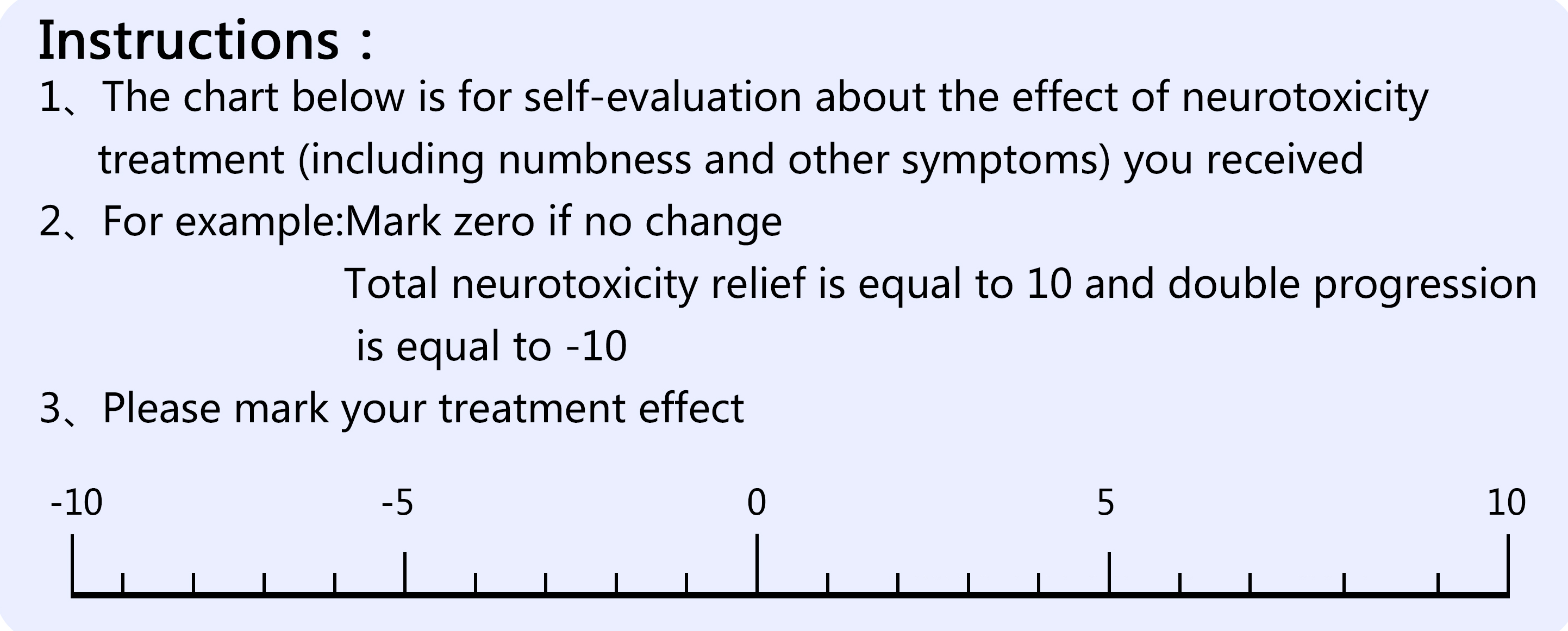


# Fig S1. Administration design


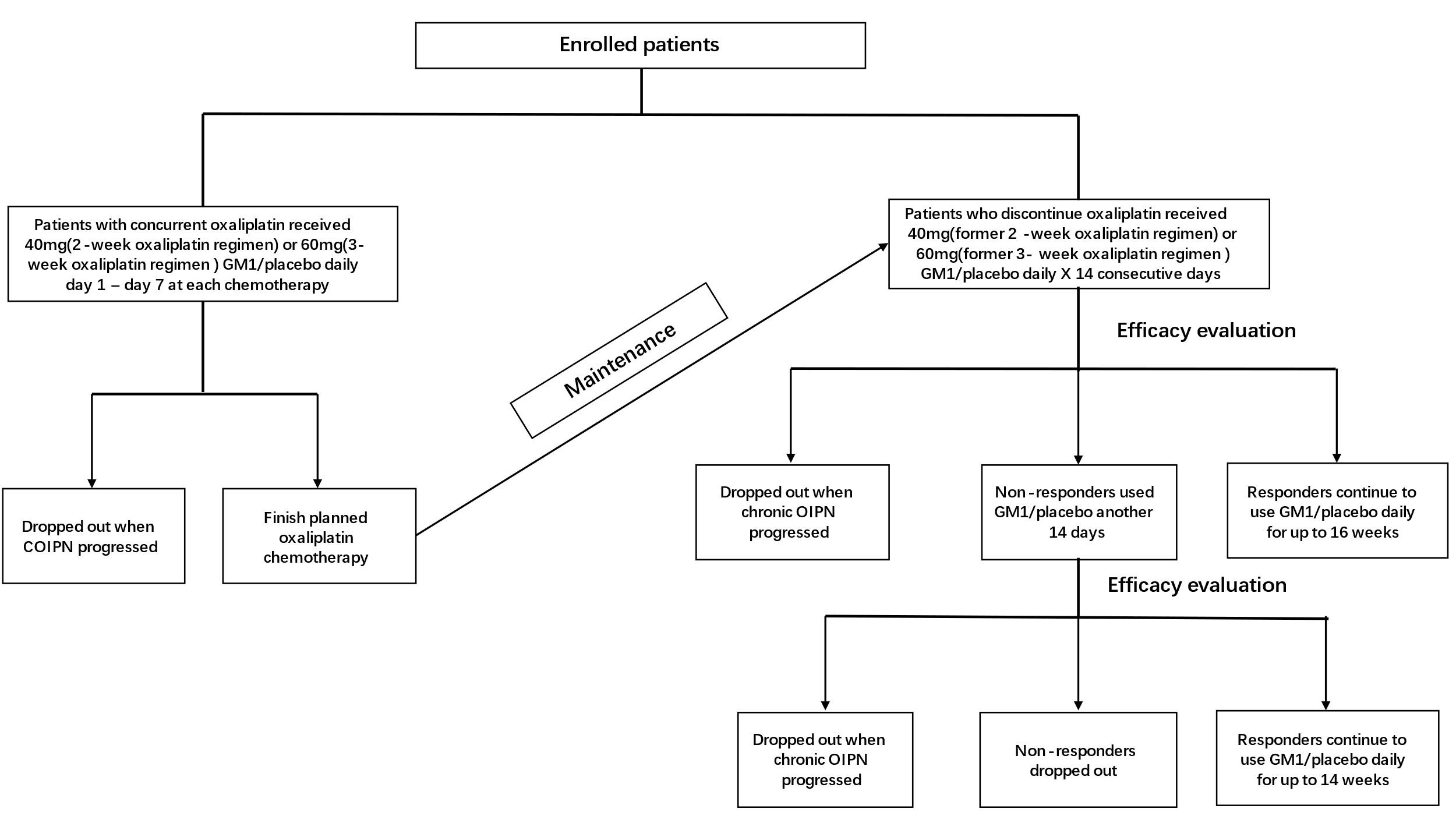


OIPN, oxaliplatin-induced peripheral neurotoxicity; GM1, monosialotetrahexosylganglioside; Responders were defined as patients who experienced 30% improvement of modified chemotherapy induced peripheral neuropathy questionnaire (MCIPN); Chronic OIPN progression was defined as MCIPN scores or VAS (visual analogue scale) showed ≥ 30% deterioration.

Fig S2. Close correlation between MCIPN and SF-36 at baseline (145 samples)
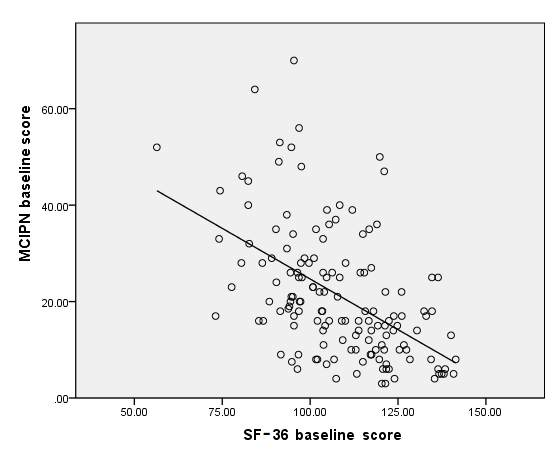


MCIPN, modified chemotherapy induced peripheral neuropathy questionnaire; SF-36, the medical outcomes study item short from health survey

Fig S3. Responders in oxaliplatin concurrent user after each treatment cycle


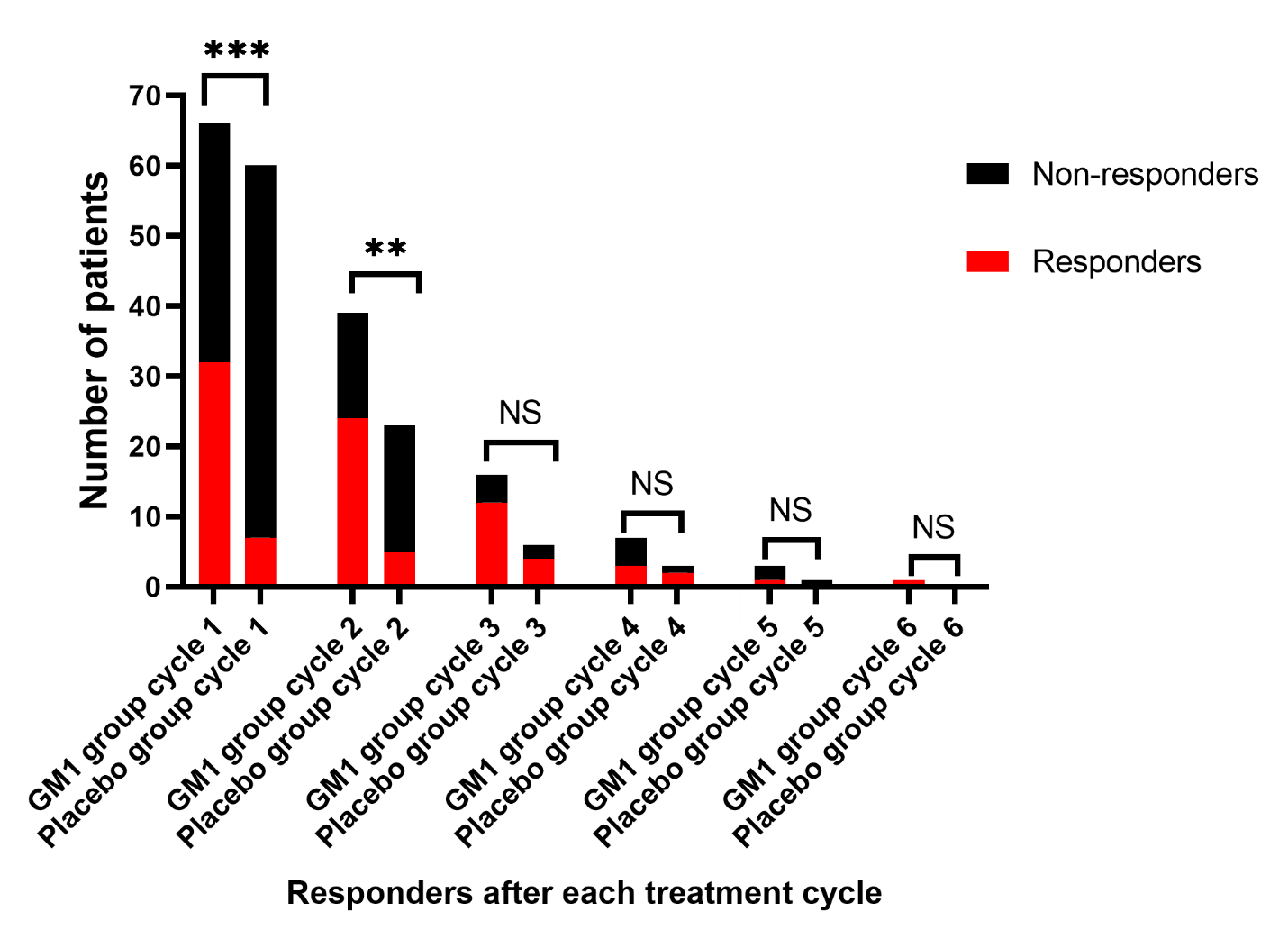


Responders was defined as patients who experienced 30% improvement in modified chemotherapy induced peripheral neuropathy questionnaire (MCIPN).

*** denotes *P* value <0.001, ** *P*=0.002 and NS, not statistically significant.

Fig S4. Responders in oxaliplatin concurrent user with accumulative treatment cycles


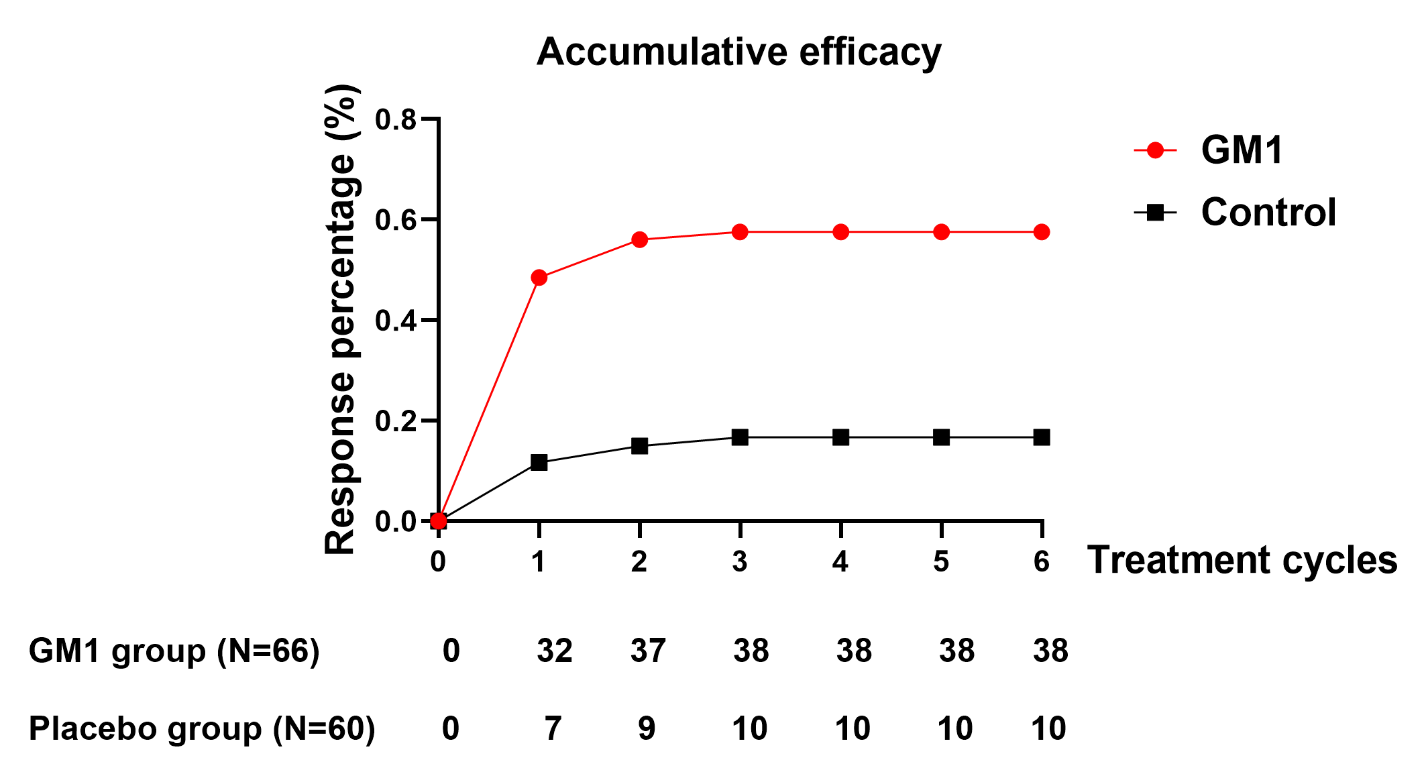


***P* value <0.0001**

Fig S5. The improved scores of each MCIPN items


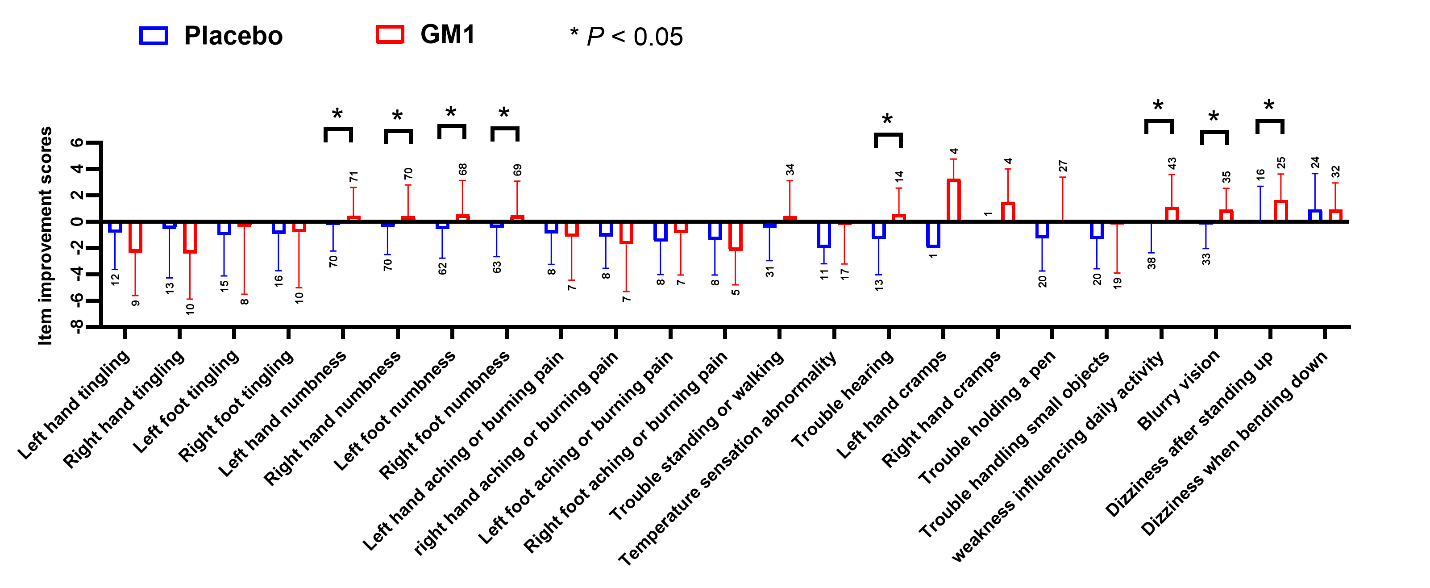


# Table S1. Efficacy of GM1 for chronic OIPN in concurrent oxaliplatin users

| Arm /Measure | MCIPN Responder | VAS Responder | Double Responder^a^ | High Responder^b^ |
| --- | --- | --- | --- | --- |
|  | n (%) | n (%) | n (%) | n (%) |
| GM1 (N=66) | 34(52) | 30(45) | 25(38) | 21(32) |
| Placebo (N=60) | 7(12) | 13(22) | 4(7) | 6(10) |
| RR | 4.34 | 2·10 | 5·59 | 3·13 |
| 95% CI | 2·08-9·04 | 1·21-3·63 | 2·06-15·12 | 1·36-7·22 |
| *P* value | <0·0001 | 0·008 | 0·0007 | 0·008 |

OIPN, oxaliplatin-induced peripheral neurotoxicity; GM1, monosialotetrahexosylganglioside; RR, risk ratio; CI, confidence interval; MCIPN, modified chemotherapy induced peripheral neuropathy questionnaire; VAS, visual analogue scale.

^a^Double responders were defined as patients who experienced 30% of both MCIPN and VAS.

^b^Responders and high responders were defined as patients who experienced 30% and 50% improvement, respectively.

# Table S2. Efficacy of GM1 for chronic OIPN in post oxaliplatin users

| Arm /Measure | MCIPN Responder | VAS Responder | Double Responder^a^ | High Responder^b^ |
| --- | --- | --- | --- | --- |
|  | n (%) | n (%) | n (%) | n (%) |
| GM1 (N=7) | 5(71) | 6(86) | 5(71) | 4(57) |
| Placebo (N=12) | 3(25) | 3(25) | 1(8) | 3(25) |
| RR | 2.86 | 3·43 | 8·57 | 2·29 |
| 95% CI | 0·96-8·47 | 1·23-9·56 | 1·24-59·30 | 0·71-7·37 |
| *P* value | 0·06 | 0·02 | 0·03 | 0·17 |

OIPN, oxaliplatin-induced peripheral neurotoxicity; GM1, monosialotetrahexosylganglioside; RR, risk ratio; CI, confidence interval; MCIPN, modified chemotherapy induced peripheral neuropathy questionnaire; VAS, visual analogue scale.

^a^Double responders were defined as patients who experienced 30% of both MCIPN and VAS.

^b^Responders and high responders were defined as patients who experienced 30% and 50% improvement, respectively.

# GM1 instruction

Monosialotetrahexosylganglioside sodium injection (Qilu Pharmaceutical Co., Ltd.)

| Approved on: April 22, 2007  [Common name]  Monosialotetrahexosylganglioside sodium injection  [Trademark name]  Shenjie  [English name]  Monosialotetrahexosylganglioside Sodium Injection  [Ingredients]  The main ingredient of this product is monosialotetrahexosylganglioside sodium. Its chemical name is monosialotetrahexosylganglioside sodium. It is a substance extracted from pig brain and has effect on nerve cell function damage.  The chemical structure formula is as follows  Molecular formula: C_73_H_130_N_3_NaO_31_ or C_75_H_134_N_3_NaO_31_  Molecular weight: 1568.84 or 1597.18  Excipient: disodium hydrogen phosphate; Sodium dihydrogen phosphate; Sodium chloride; Water for injection.  Monosialotetrahexosylganglioside sodium  [Character]  This product is a colorless clear liquid, sometimes with slight opalescence.  [Indications]  It is used to treat vascular or traumatic central nervous system injury; Parkinson's disease.  [Drug specifications  （1）2ml：20mg；（2）2ml：40mg；（3）5ml：100mg  [Usage and dosage]  20-40 mg daily, intramuscular injection or slow intravenous drip once or several times according to the doctor's advice.  In the acute stage of lesions (especially acute trauma): 100 mg per day, intravenous drip; After 2-3 weeks, it was changed to maintenance dose, 20-40 mg per day, generally for 6 weeks. For Parkinson's disease, the first dose was 500-1000 mg, intravenous drip; From the second day, 200mg daily, subcutaneously, intramuscularly or intravenously, generally for 18 weeks.  [Adverse reactions]  A small number of patients with skin rash reaction after using this product should be advised to discontinue.  [Taboo]  The product is forbidden under the following conditions: it has been proved allergic to the product; Genetic glycolipid metabolism is abnormal (ganglioside accumulation disease, such as familial dementia and retinodegeneration).  [Precautions]  Please read the instructions carefully before using this product; It should be used according to the doctor's advice.  [Medication for pregnant and lactating women]  According to the literature, in the experimental animals, no adverse reactions were reported when monosialotetrahexosylganglioside was used during pregnancy and lactation.  [Medication for children]  So far, no adverse reactions have been reported in children.  [Medication for the elderly]  So far, no adverse reactions have been reported in elderly patients.  [Drug interaction]  unclear.  [Drug overdose]  Up to now, there is no report of overdose. The daily dose of 1000mg was still well tolerated.  [Pharmacology and toxicology]  Pharmacological effect: monosialotetrahexosylganglioside can promote the function recovery of central nervous system injury caused by various reasons. The mechanism of action is to promote "neuroplasticity" (including the survival of nerve cells, axon growth and synaptic growth). The monosialotetrahexosylganglioside has protective effect on the secondary nerve degeneration after injury. monosialotetrahexosylganglioside has a positive effect on the hemodynamic parameters of the brain and the brain edema after injury. The activity of membrane enzyme was improved by monosialotetrahexosylganglioside to reduce the edema of nerve cells. Animal experiments showed that monosialotetrahexosylganglioside could improve the behavior disorder caused by Parkinson's disease.  Toxicology: the literature shows that LD50 of monosialotetrahexosylganglioside is 872mg/kg (i.v.) to > 8g/kg (s.c.), depending on the animal species and the route of administration. The subacute and chronic toxicity tests, teratogenesis tests, reproductive toxicity tests, perinatal toxicity tests and mutagenic tests conducted by various animals did not show any toxic effects of the product.  [Pharmacokinetics]  Exogenous monosialotetrahexosylganglioside can bind with the nerve cell membrane in a stable way, which causes the function change of the membrane. The peak of radioactivity was measured in the brain and spinal cord 2 hours after administration. Halve in 4-8 hours. The drug is removed slowly, mainly through the kidney.  [Storage]  Sealed and stored at room temperature (15-30 ℃).  [Packaging]  Ampoule package, 5 pieces / box (2ml: 20mg; 2ml：40mg）； 1 piece / box (5ml: 100mg).  [Validity period]  24 months.  [Executive standard]  YBH19592004（2ml：20mg）  YBH20922005（2ml：40mg； 5ml：100mg）  [Approval number]  H20046213  H20056782  H20056783  [Manufacturer]  Qilu Pharmaceutical Co., Ltd |
| --- |
